# Authors’ Reply to: And Justice for All? There Is More to the Interoperability of Contact Tracing Apps Than Legal Barriers. Comment on “COVID-19 Contact Tracing Apps: A Technologic Tower of Babel and the Gap for International Pandemic Control”

**DOI:** 10.2196/26630

**Published:** 2021-05-26

**Authors:** Li Du, Vera Lúcia Raposo, Meng Wang

**Affiliations:** 1 Faculty of Law University of Macau Macau China; 2 Faculty of Law University of Coimbra Coimbra Portugal

**Keywords:** COVID-19, contact tracing, data protection, privacy, interoperability, global health, public health

We appreciate Crutzen’s comments [1] on our analysis [2] of the technologic Tower of Babel, which analyzed the global functioning and interaction of COVID-19 contact tracing apps.

In his critique, Crutzen argued that it is not all about the law, but also about ethics. We agree with this proposition. Ethics and the principles of digital governance are essential to structure the digital world [3], especially during (and after) a pandemic [4].

We also agree with his observation that the eHealth Network has contributed to achieving the interoperability guidelines for contact tracing apps within the European Union (EU). However, the utility of this network is limited to Europe. Because member states share a basic framework regarding data protection (ie, the General Data Protection Regulation [GDPR]), unsolved legal conflicts between national laws on privacy are not likely to happen. Without such a common ground for data protection, the Google-Apple API (application programming interface) code can only help governments around the world to fast track the adoption of contact tracing app technology, but it may not solve the interoperability of contact tracing apps between countries (eg, between an EU member state and the United States). Moreover, European countries are more willing to work together in this regard and to adapt their national laws and technical practices, as it has already happened within several other domains: passports, border controls, currency, VAT (value-added tax) rates, and roaming. These are all areas related to the free circulation of people that EU member states have agreed to “harmonize” in order to facilitate free movement and a single European market [5]. Guidelines for interoperability between contact tracing mobile apps are just another step toward achieving that goal. On page 6, we underlined that “a broader consensus is required for the international community, since a common European approach will only solve the problem within the European Union.”

In general, we are concerned with both the legal and ethical disconformity between different apps, while Crutzen focuses primarily on ethical concerns and the protection of human rights. In a sense, the two papers appear complementary, rather than to be in conflict with each other.

We take issue with one of Crutzen’s statements, specifically the idea that making a contact tracing app mandatory for international travelers is unacceptable. In this case, we would need to abolish several other requisites imposed on international travelers, such as visa requirements for certain destinations or even the mere presentation of passports and other traveling documents. The most paradigmatic example is the proof of immunization required for some destinations, which, like the mandatory contact tracing app, is required for public health reasons and supported by institutions such as the World Health Organization [6]. Under Crutzen’s reasoning, we would have to conclude that vaccination requirements are unethical and constitute a violation of travelers’ rights.

Moreover, many countries are still requiring a 14-day compulsory quarantine for international travelers [7]. Using the contact tracing app for international travelers would actually make international travel easier, giving people more freedom for traveling during the pandemic. Even if the mandatory use of contact tracing apps can be considered burdensome, it would facilitate the prevention of more severe intrusions in travelers’ personal freedom.

A contact tracing app that is mandatory for international travelers is a threat to fundamental rights. We recognize that. However, in light of the delicate balance between public health and individual rights and freedoms, and considering that from the various restrictions that can be imposed, we believe this measure is fully acceptable, especially based on an assessment of necessity, proportionality, and adequacy. It is certainly more acceptable than the ban on international traveling still in place in many parts of the world.

## Abbreviations

API: application programming interface
EU: European Union
GDPR: General Data Protection Regulation
VAT: value-added tax
